# F153. NEUROMAGNETIC MISMATCH NEGATIVITY IN CLINICAL HIGH RISK AND FIRST-EPISODE PSYCHOSIS INDIVIDUALS

**DOI:** 10.1093/schbul/sby017.684

**Published:** 2018-04-01

**Authors:** Emmi Mikanmaa, Marc Recasens, Hanna Thuné, Tineke Grent-’t-Jong, Andrew Gumley, Matthias Schwannauer, Stephen Lawrie, Ruchika Gajwani, Joachim Gross, Peter Uhlhaas

**Affiliations:** 1 University of Glasgow; 2 University of Edinburgh; 3 Royal Edinburgh Hospital

## Abstract

**Background:**

Auditory mismatch negativity (MMN) and its magnetic counterpart (MMNm) are event-related potentials/fields elicited by violations of previously established auditory regularities (Näätänen et al. 2007). Reduced MMN amplitude is a robust finding in both medicated and unmedicated schizophrenia (ScZ) patients (for a review, see Umbricht & Krljes, 2005), potentially indicating impaired predictive processes (Sauer et al. 2017). Furthermore, several studies have examined MMN responses in individuals at clinical high risk for psychosis (CHR) and have found MMN deficits to be present already in this population, indicating that MMN is compromised before psychosis onset and could represent a marker of risk for psychosis development. However, not all studies have reported significant differences in MMN amplitude between clinical high risk and healthy individuals and there is evidence to suggest that MMN deficits are primarily observed in CHR individuals who transition to psychosis (Bodatsch et al., 2015).

**Methods:**

The purpose of the present study is to investigate MEG recorded MMNm amplitude responses to both duration deviants and sound omissions in n = 90 CHR-individuals who were screened with the Schizophrenia Proneness Instrument and the Comprehensive Assessment of At Risk Mental States. N = 50 healthy controls served as a comparison group. We employed an auditory oddball paradigm in which three different sequences of auditory stimuli were presented binaurally at ~ 70 dB (150 ms SOA, 700 – 100 ms ISI). The standard sequence contained five identical tones (80 ms) and the deviant sequence contained four identical tones and one duration deviant tone (40 ms). In addition, an omission sequence which contained only four identical tones was included to examine auditory predictions. Participants were instructed to focus their attention away from the sounds and to perform a simple visual detection task. We analyzed MMNm responses to unpredictable deviant and omitted sounds in bsensor and source space. The linearly constrained minimum variance (LCMV) beamformer was used to identify the generators of the MMNm responses and to compute artefact-free source-level time-courses.

**Results:**

Consistent with previous studies revealing MMN sources in auditory cortical areas, bilateral MMNm sources were localized in auditory cortices for both duration deviants and sound omissions. Our data show attenuated MMNm responses elicited by duration deviant sounds in CHR individuals compared to healthy controls.

**Discussion:**

Taken together, the study findings suggest that reduced MMNm amplitude is present before psychosis onset and reflects aberrant predictive processing rather than deficient stimulus-specific adaptation. Clinical follow-up measurements will be obtained to determine whether MMNm amplitudes are a potential biomarker for predicting onset of psychosis.

